# Cellular uptake and in vitro antitumor efficacy of composite liposomes for neutron capture therapy

**DOI:** 10.1186/s13014-015-0342-7

**Published:** 2015-02-22

**Authors:** Tanja Peters, Catrin Grunewald, Matthias Blaickner, Markus Ziegner, Christian Schütz, Dorothee Iffland, Gabriele Hampel, Thomas Nawroth, Peter Langguth

**Affiliations:** Institute of Pharmacy and Biochemistry, Department of Biopharmaceutics and Pharmaceutical Technology, Johannes Gutenberg University Mainz, Staudingerweg 5, D-55128 Mainz, Germany; Institute of Nuclear Chemistry, Johannes Gutenberg University Mainz, Fritz-Strassmann Weg 6, D-55128 Mainz, Germany; AIT Austrian Institute of Technology, Health & Environment Department, Biomedical Systems, Donau-City-Strasse 1/2, A-1220 Vienna, Austria

**Keywords:** Gadolinium, Liposomes, Drug uptake, Neutron capture therapy, Glioma, Theranostic

## Abstract

**Background:**

Neutron capture therapy for glioblastoma has focused mainly on the use of ^10^B as neutron capture isotope. However, ^157^Gd offers several advantages over boron, such as higher cross section for thermal neutrons and the possibility to perform magnetic resonance imaging during neutron irradiation, thereby combining therapy and diagnostics. We have developed different liposomal formulations of gadolinium-DTPA (Magnevist®) for application in neutron capture therapy of glioblastoma. The formulations were characterized physicochemically and tested in vitro in a glioma cell model for their effectiveness.

**Methods:**

Liposomes entrapping gadolinium-DTPA as neutron capture agent were manufactured via lipid/film-extrusion method and characterized with regard to size, entrapment efficiency and in vitro release. For neutron irradiation, F98 and LN229 glioma cells were incubated with the newly developed liposomes and subsequently irradiated at the thermal column of the TRIGA reactor in Mainz. The dose rate derived from neutron irradiation with ^157^Gd as neutron capturing agent was calculated via Monte Carlo simulations and set in relation to the respective cell survival.

**Results:**

The liposomal Gd-DTPA reduced cell survival of F98 and LN229 cells significantly. Differences in liposomal composition of the formulations led to distinctly different outcome in cell survival. The amount of cellular Gd was not at all times proportional to cell survival, indicating that intracellular deposition of formulated Gd has a major influence on cell survival. The majority of the dose contribution arises from photon cross irradiation compared to a very small Gd-related dose.

**Conclusions:**

Liposomal gadolinium formulations represent a promising approach for neutron capture therapy of glioblastoma cells. The liposome composition determines the uptake and the survival of cells following radiation, presumably due to different uptake pathways of liposomes and intracellular deposition of gadolinium-DTPA. Due to the small range of the Auger and conversion electrons produced in ^157^Gd capture, the proximity of Gd-atoms to cellular DNA is a crucial factor for infliction of lethal damage. Furthermore, Gd-containing liposomes may be used as MRI contrast agents for diagnostic purposes and surveillance of tumor targeting, thus enabling a theranostic approach for tumor therapy.

## Background

Neutron capture therapy (NCT) is a cancer treatment approach based on accumulation of neutron capture agent at the tumor site and irradiation of the tumor with thermal neutrons as a second step. In the past, research has focused primarily on ^10^B as neutron capture agent. However, ^157^Gd may be an alternative element for NCT [1]. Gadolinium provides several advantages over boron, namely the highest cross section for thermal neutrons known for stable elements (^157^Gd: 255,000 barn) and following administration, the possibility to trace the agent through the body via magnetic resonance imaging (MRI), thereby allowing a theranostic approach to cancer treatment [2]. The fission products of ^10^B, an alpha particle and recoiling Li-nucleus, have path lengths of approximately 9–12 μm, i.e. the range of an individual cell. In contrast, neutron capture reaction of ^157^Gd results in the generation of ^158^Gd, at least five Auger- and inner conversion electrons and photons of different energies. Auger- and inner conversion electrons are thought to be the main contributors to the cell killing effect of Gd-NCT. It was shown by Martin and co-authors 1989 [3] that the Auger electrons from Gd-neutron capture reaction led to DNA double-strand breaks and subsequent cell death. Since path lengths for Auger and inner conversion electrons are extremely short (nm to lower μm range), intracellular accumulation and distribution of Gd is very important for the radiation effect. To reach the main target for radiation therapy in cells, the DNA, the Gd-atom has to be located in close proximity to the cellular nucleus. In addition to the short-ranged Auger and inner conversion electrons, the ^157^Gd neutron capture reaction produces long-range gamma rays of different energies up to 8 MeV whose flight ranges are not limited to a single cell. Similar to photon radiation therapy, these gamma rays may also interact with cellular structures if gadolinium was located outside the target cells, thus inflicting DNA lesions, however to a much smaller extent. Furthermore, an additional dose may be produced by self-absorption of the gamma rays by the gadolinium load at the tumor site, thus generating further Auger electrons via photoelectric effect.

A crucial point in gadolinium neutron capture therapy is the provision and retention of high gadolinium concentration in the target tissue. Shih and Brugger 1992 [4] calculated that 50–200 μg ^157^Gd/g wet tumor tissue should be sufficient for successful cancer treatment. The administration of liposomes as drug carrier systems offers several advantages over the free drug, such as the shielding of entrapped drugs from degradation and targeting the drug carrier exclusively to the tumor site by addition of cell-specific targeting structures. Furthermore, the uptake of liposomes may supersede the uptake of free drug due to higher payload of the carrier and by taking advantage of different uptake mechanisms.

In the present study, we introduce several novel Gd-containing liposomal formulations for application in neutron capture therapy. Composite liposomes employing different lipids were designed in order to facilitate uptake into cancer cells and to deliver sufficient amounts of Gd into the target cell. Lipids were chosen according to their physicochemical and biogenic characteristics or estimated uptake properties, e.g. anionic Cardiolipin, a component of mitochondrial membrane which is involved in the mitochondria-dependent apoptosis [5] and fusogenic DOPE, known for the ability to escape endosomal compartments after uptake through conformational change [6-8]. Cationic DOTAP ensures electrostatic attraction between cellular membranes and liposomes, thus facilitating binding as the first step in the liposomal drug uptake process [9,10]. As a specified targeting structure, folate, a substrate for folate receptor alpha, which is overexpressed in many tumor types, was selected. While reduced folate carrier (RFC) is ubiquitous, FR alpha (FR α) is rarely expressed in normal tissues, but known for its overexpression in tumor tissues, amongst other in breast and ovarian cancer, melanoma and brain tumors such as glioma [11-15].

The specific aim of this study encompasses an evaluation of the suitability of Magnevist-entrapping composite liposomes as nanocarrier system for Gd-NCT of glioma cells. Liposomes were characterized physicochemically and according to their toxicity and uptake into F98 (rat glioma) and LN229 (human glioblastoma) cells. Performance of the drug carrier system for neutron capture agents was investigated in a glioma cell model regarding drug uptake and cell survival subsequent to irradiation with thermal neutrons. Cell survival was then related to Gd-concentration in cells. Simultaneously, Monte Carlo simulations of the mixed neutron-gamma field and the resulting dose on the 96 well plates in the medium and on the cellular level were performed to assess the influence of Gd-concentration and -location on cell survival.

## Methods

### Liposome preparation

Liposomes were prepared according to the lipid film-extrusion method. Lipids were purchased from Avanti Polar Lipids, Alabaster, AL, USA (folate-PEG2000-DSPE), Sigma Aldrich Co., St. Louis, MO, USA (DOPC, cholesterol, Cardiolipin) or were a gift from Lipoid GmbH, Ludwigshafen, Germany (DOPE, DOTAP). Lipid mixtures consisted of (mol %): DOPC: cholesterol: DOPE (70:20:10), DOPC: cholesterol: Cardiolipin (70:20:10), DOPC: cholesterol: DOTAP (57.41:33.35:9.23), DOPC: cholesterol: folate-PEG2000-DSPE (63.19:36.67:0.13), DOPC: DOPE (50:50). A mixture of the required lipids dissolved in chloroform was dried under vacuum on a rotary evaporator to form a thin lipid film. Subsequently, the film was rehydrated with Magnevist® solution (Bayer Vital GmbH, Leverkusen, Germany) or PBS (Life Technologies GmbH, Darmstadt, Germany) to obtain a lipid concentration of 40 mg/ml. The mixture was vortexed thoroughly and subjected to five freeze-thaw cycles, i.e. the dispersion was frozen in liquid nitrogen for one minute and thawed in a water bath of 37°C for five to six minutes. The resulting lipid dispersion was extruded eleven times through a 100 nm polycarbonate membrane using an Avanti Mini Extruder® (Avanti Polar Lipids, Alabaster, AL, USA). Non-entrapped material was removed via minicolumn centrifugation/gel permeation chromatography method (GPC) with Sephadex™ G-25 Medium (GE Healthcare, Uppsala, Sweden) as column material.

### Liposome characterization

Size measurements were performed via dynamic light scattering with Zetasizer Nano ZS (Malvern Technologies, Herrenberg, Germany) at 173° scattering angle. Liposome dispersions were diluted with PBS to yield a lipid concentration of 0.25 mg/ml. Determination of zeta potential was carried out with the same instrument, with liposomes suspended in double-distilled water at a lipid concentration of 0.25 mg/ml.

Entrapment efficiency was determined via mass-spectrometric analysis of Gd-content in liposome dispersions after GPC in regard to total Gd-amount of the liposome dispersion before GPC treatment.

### In vitro release experiments

Release of Magnevist from liposomes was tested for DOPC-DOPE and DOTAP-liposomes via flow-through dissolution in a Sotax CE 7 apparatus with liposome adapter devices (Sotax AG, Allschwill, Switzerland). Adapters were used with a cellulose ester dialysis membrane MWCO 3.5 to 5 kD (Spectrum Laboratories, Rancho Dominguez, CA, USA). 450 μl of liposome dispersion or diluted Magnevist solution were pipetted into adapters and placed into a 22.6 mm tablet flow-through cell. To ensure laminar flow, glass beads were filled into the base of each dissolution cell. Dissolution medium consisted of 50 ml PBS in a closed circulation, with a pump rate of 16 ml/min. Dissolution was performed for 3 h at 37°C. Samples of 1 ml volume were extracted at defined time points and replenished with fresh medium. Gadolinium content of samples was determined via ICP-MS.

## Cell culture

### Irradiation experiments

F98 rat glioma cell line and LN229 human glioblastoma cell line were purchased from LGC/American Type Culture Collection, Middlesex, UK. Two days prior to radiation experiments, cells were seeded into 96 well plates at a density of 4000 cells per well. Medium was exchanged against fresh growth medium supplemented with liposomal formulations at concentrations of 0.27 to 0.47 mg Gd/ml or Magnevist solution three hours prior to neutron irradiation. Cell culture plates were then sealed with adhesive tape to prevent loss of CO_2_, transported to TRIGA Mark II reactor and always stacked in the same order into a polyethylene irradiation box. Neutron irradiation was carried out at room temperature with neutron fluences of about 2.4 to 2.7 · 10^12^ particles per cm^2^ and photon fluences of about 6.3 · 10^11^ to 7.2 · 10^11^ particles/cm^2^ corresponding to 15 min irradiation with 20 kW reactor power and about 3.6 to 4.05 · 10^12^ particles/cm^2^ and photon fluences of about 9.45 to 10.8 · 10^11^ particles/cm^2^ corresponding to 22.5 min irradiation with 20 kW reactor power, respectively, at the central irradiation chamber of the thermal column. After irradiation and a cooling down period of approximately 45 min, the activated medium containing liposomes or Magnevist® was aspirated off and replenished with fresh growth medium. Cells were kept under recommended growth conditions, until MTT proliferation assay was performed.

### Uptake experiments

For uptake experiments, cells were seeded into 100 mm petri dishes at densities of 1.4 · 10^6^ and 1.6 · 10^6^ cells for F98 and LN229 cells, respectively. After two days under recommended growth conditions, medium was changed and cells were incubated with liposomal formulations or Magnevist solution for 1 to 24 hours. Cells were then washed thoroughly with ice-cold PBS, harvested and counted for mass spectrometric analysis of Gd-content.

### MTT proliferation assay

MTT (3-(4,5-Dimethylthiazolyl-2)-2,5-diphenyl-2H-tetrazoliumbromide, Carl Roth GmbH & Co. KG, Karlsruhe, Germany) cell proliferation assays were performed 97 h after cell irradiation experiments to estimate cell survival. Cell growth medium was aspirated off, replaced with 100 μl of 0.5 g/l MTT solution and incubated for 30 min. In the next step, MTT working solution was removed and exchanged against 180 μl dimethyl sulfoxide (Carl Roth GmbH & Co. KG, Karlsruhe, Germany) which were also left to incubation for 30 min at 37° to ensure complete dissolving of the formazan crystals. The resulting blue colored extract was pipetted into a fresh microplate for the read-out in a Tecan infinite™ F200 plate reader (Tecan Group Ltd., Männedorf, Switzerland) at absorption wavelength 560 nm and reference wavelength 690 nm.

For toxicity tests, MTT assay was performed as follows: cells were incubated with liposomal formulations or Magnevist for three hours in parallel to irradiation experiments. These cells were treated in the same way as irradiated cells, i.e. concerning incubation time with liposomal formulations, transport to the TRIGA reactor and medium exchange, except for the neutron irradiation. Repetitive MTT tests were then performed every 24 hours for three days. Results are arithmetic means ± SD of all three assays, based on untreated control cells in growth medium.

### Analysis of cellular Gd-content

Gd-content in liposome- or Magnevist®-treated cells was determined via inductively coupled plasma mass spectrometry. Briefly, cells harvested from uptake experiments were digested with hydrogen peroxide/nitric acid mixture and diluted with double distilled water. After addition of 10 ppm Eu as internal standard, the solution was analyzed on an Agilent ICP-MS 7500 ce (Agilent Technologies, Santa Clara, CA, USA), using a concentric 0.05 ml/min nebulizer and a cyclone spray chamber. Rinsing between samples was done with solutions consisting of i) (1.25% w/w) HNO_3_ and ii) (1.25% w/w) nitric acid. All chemicals were purchased from Carl Roth GmbH and Co. KG, Karlsruhe, Germany and were of analytical grade or higher.

### Monte Carlo simulations

The neutron and photon dose during irradiation experiments were calculated using MCNP 5 program (Los Alamos National Security, Los Alamos, New Mexiko, USA). The neutron field was simulated with a defined source plane at the front of the thermal column. The agreement of the model with the real conditions at TRIGA reactor had been confirmed before [16,17]. The geometry of the well plates was incorporated into the MCNP model alongside with the gadolinium concentration of the different wells which was taken from the analysis mentioned in the previous paragraphs. The natural abundance of gadolinium isotopes was used as isotopic composition. In the simulations, the optional libraries rmccsa and misc5xs were used in order to account for the gamma production following the neutron capture by gadolinium. Different MCNP transportation modes were applied such as n (neutron tracking only, local dose deposition of others), np (neutron and photon tracking, local dose deposition of others) and npe (neutron, photon and electron) tracking. Different MCNP tallies were set and for comparison also the Kerma approximation was applied where the entire energy released in a nuclear reaction is deposited locally, i.e. reaction rate times the released energy of the respective reaction.

## Results

### Liposome characterization

Liposomal formulations of Magnevist showed narrow size distributions with mean particle sizes of approximately 136 to 152 nm. The introduction of charged or functionalized lipids did not affect liposome size significantly as shown in Table 1. As expected, zeta potential measurements revealed high influence of charged lipids in the lipid mixture, leading to high positive zeta potential of DOTAP-containing liposomes and high negative zeta potential of Cardiolipin-containing liposomes. Entrapment efficiencies of the Gd-chelate complex were comparable for all tested liposome compositions (9.4 to 10.2%) except for the slightly lower entrapment in Cardiolipin-containing formulation (6.6%). Concentration of gadolinium in the final liposome suspension was therefore 3.3 to 5.9 mg/ml, respective Gd-concentrations per well for irradiation experiments are given in Table 1.

**Table 1:** **Size, zeta potential and entrapment efficiency for Magnevist in liposomal formulations**

**Formulation (mol%)**	**Diameter [nm] ± SD**	**Zeta potential [mV] ± SD**	**Encapsulation efficiency [%]**	**Gd concentration per well [mg/ml]**
DOPC-Chol-DOTAP (57:33:9)	136.20 ± 8.07	41.25 ± 2.49	10.24 ± 4.85	0.47
DOPC-Chol-CL (70:20:10)	152.11 ± 10.22	−67.77 ± 2.70	6.56 ± 1.74	0.27
DOPC-Chol-DOPE (70:20:10)	133.19 ± 5.76	−15.53 ± 7.53	9.37 ± 2.83	0.36
DOPC-Chol-FolPEG 2000 (63:37:0.13)	136.77 ± 8.81	−24.70 ± 3.10	9.60 ± 1.75	0.36
DOPC-DOPE (50:50)	138.54 ± 10.17	−17.78 ± 6.04	10.21 ± 2.53	0.46

### Toxicity of Magnevist and liposomal Magnevist formulations

**Figure 1:**
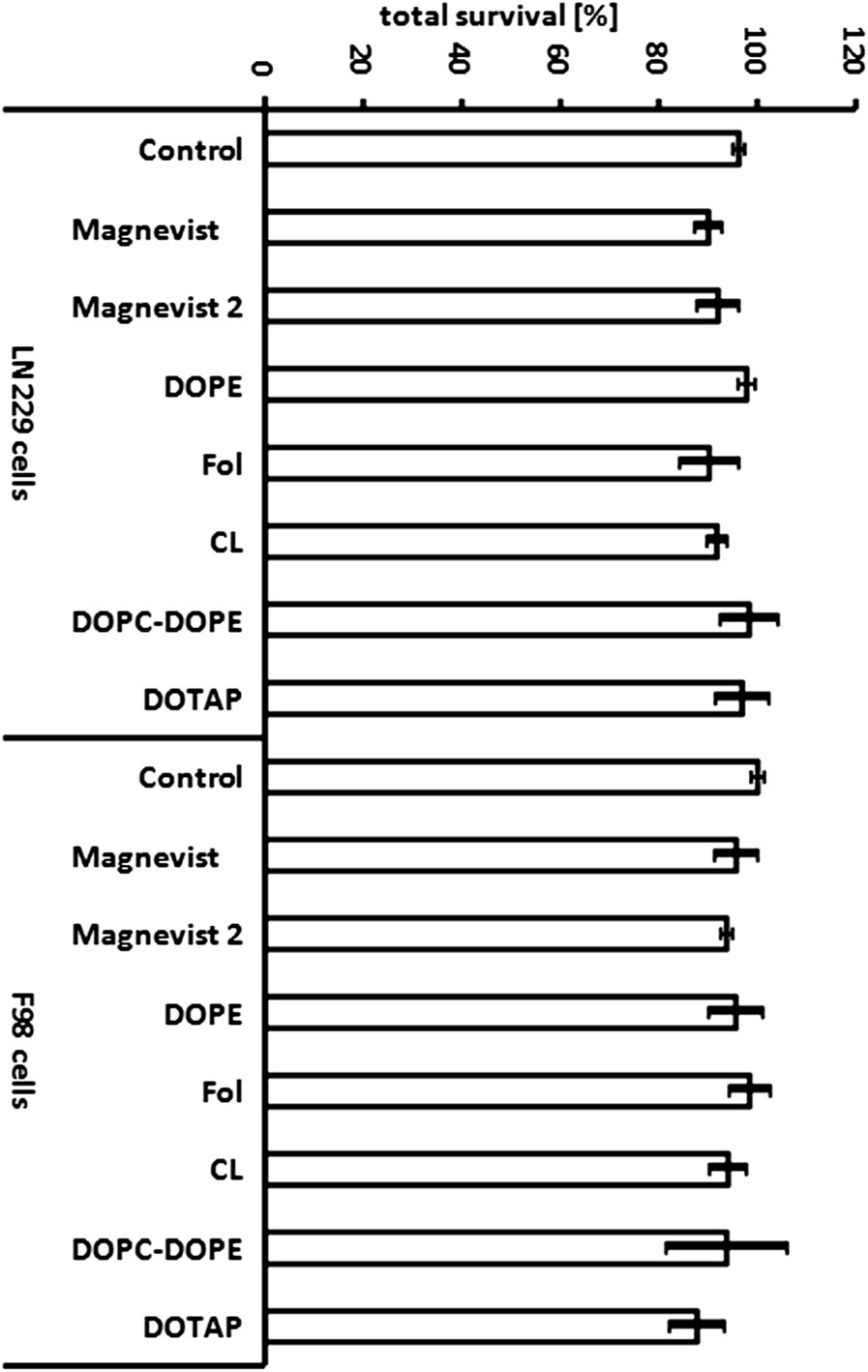
**Toxicity of Magnevist and Magnevist containing liposomal formulations.** Cells were incubated for three hours with respective liposomal Magnevist solution at lipid concentration of 4 mg/ml or with Magnevist solution. Gd-concentration of Magnevist solution was 0.34 mg/ml for ‘Magnevist’ and 0.68 mg/ml for ‘Magnevist 2’. Table 1 lists respective concentrations in wells. Cells of the control group were left in complete growth medium without supplement of liposomes or Magnevist. MTT assays were performed every 24 hours for three days subsequent to treatment. Results are arithmetic means of the three assays, based on medium control group, error bars represent SD.

Determination of cytotoxic effects of liposomal and free Magnevist was performed via MTT assay in parallel to irradiation experiments. As shown in Figure 1, Magnevist and liposomal formulations thereof showed no relevant toxicity for both cell lines. Except for cationic DOTAP-formulation with 12% decrease in cell survival of F98 cells, all liposomal preparations lead to less than 10% decrease in cell viability for both F98 and LN229 cells. The higher toxicity for the cationic formulation is probably caused by the presence of the cationic charge on the liposome surface. Lv et al. [18] concluded that cationic lipids and polymers bring about a certain cell toxicity due to the charge they carry and to their subsequent interaction with essential cellular enzymes. However, since in our case the cytotoxicity of cationic liposomes was only moderately higher than for neutral and negatively charged formulations (12% in contrast to 10%), this aspect was considered as negligible.

### In vitro release of Gd from liposomal formulations

**Figure 2:**
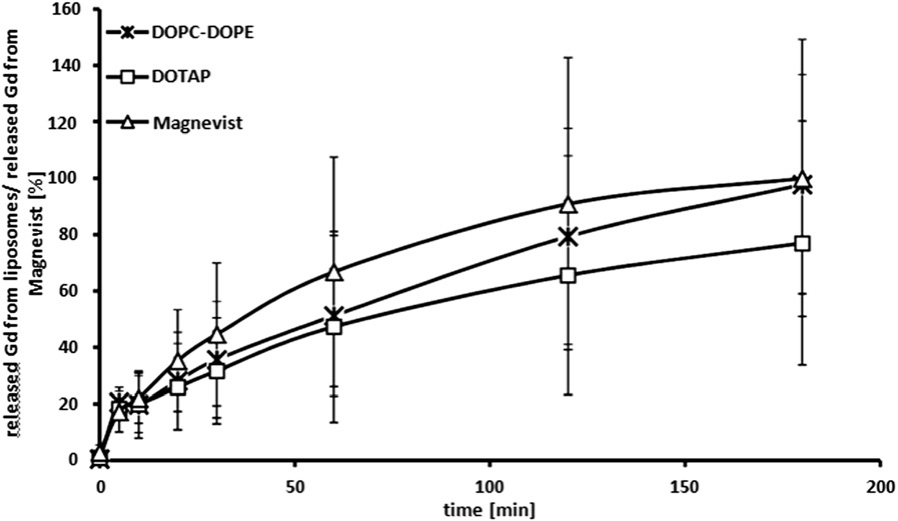
**In vitro release of gadolinium-DTPA from liposomal formulations.** Percent release of Magnevist from DOTAP- and DOPC-DOPE-liposomes. Magnevist solution alone was used as a control. Dissolution was performed for three hours at 37°C in a flow-through cell equipped with liposome adapters and in closed circulation. Results are arithmetic means, error bars represent SD, n = 3.

Release profiles (Figure 2) show an initial burst of gadolinium-DTPA from liposomal formulations in the first five minutes of the dissolution experiment. Since free Magnevist also shows this stage, the fast release apparently attributes to already liberated gadolinium-DTPA diffusing out from the dissolution bag. In the second stage, release is also relatively fast, until the amount of liberated Gd-compound asymptotically approaches a maximum after 3 hours. At this time point, the remaining gadolinium-DTPA trapped inside the liposomes is approximately 3% for DOPC-DOPE-liposomes and more than 20% for DOTAP-liposomes. After one hour, approximately 50% of the neutron capture agent is still safely entrapped in liposomal formulations. Liposomes show therefore a sufficiently high retention of gadolinium-DTPA for at least one hour at 37°C. Nevertheless, a three-hour time span was set as incubation period to ensure sufficient opportunity for cellular uptake.

### Uptake of Gd-DTPA into cells

**Figure 3:**
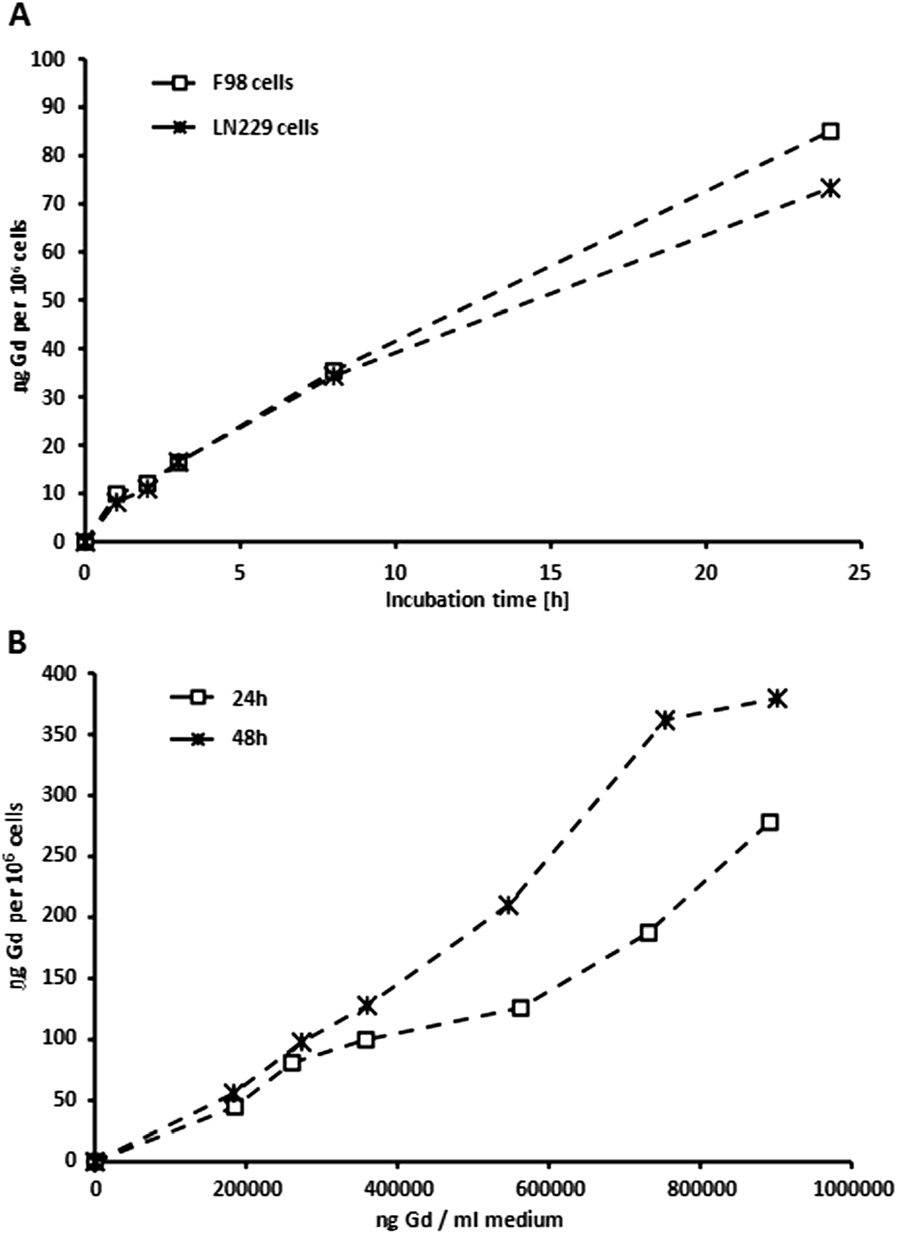
**Time- and concentration dependent uptake of free Magnevist into F98 and LN229 cells. (A)** LN229 and F98 glioma cells were incubated with Magnevist solution (0.34 mg/ml) for time periods of 1 to 24 h. **(B)** F98 cells were incubated with different concentrations of Magnevist solution (0.18 to 0.9 mg/ml) for 24 and 48 h, respectively. Resulting Gd-concentration in the cells was determined via ICP-MS, n = 3.

**Figure 4:**
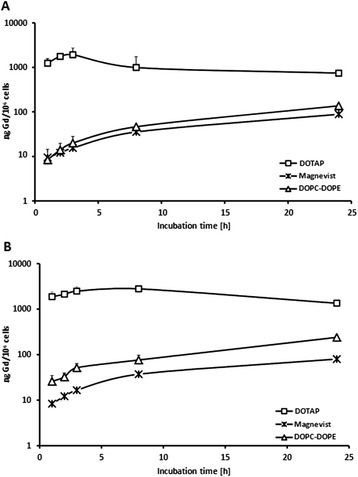
**Time-dependent uptake of liposomal gadolinium formulations (DOTAP and DOPC-DOPE) and Magnevist into F98 cells.** Gd-concentration in **(A)** F98 and **(B)** LN229 cells after incubation with liposomal Magenvist formulations, i.e. DOPC-DOPE and DOTAP (1 mg lipid/ml, respective Gd-concentration cf. Table 1) and Magnevist (0.34 mg Gd/ml) for 1 to 24 h. Gd-concentration was determined via ICP-MS. Results are arithmetic means, error bars represent SD, n = 3.

**Figure 5:**
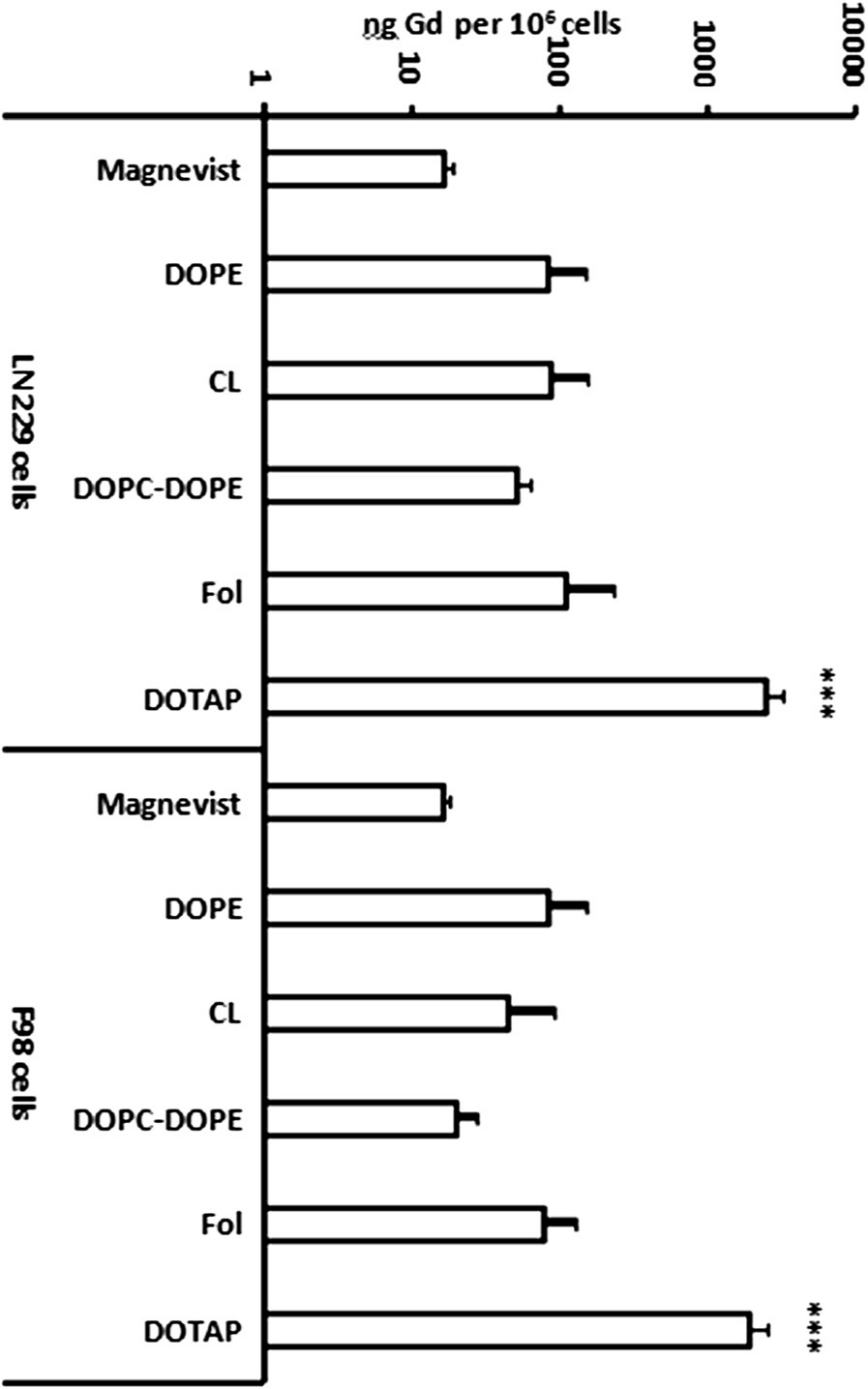
**Uptake of liposomal gadolinium-formulations and Magnevist into (right) F98 and (left) LN229 cells.** Gd-concentration (logarithmic scale) in LN229 and F98 cells after 3 h incubation with liposomal Magnevist formulations (lipid concentration 1 mg/ml, respective Gd-concentration cf. Table 1) and free Magnevist (0.34 mg Gd/ml). Gd-concentration was determined via ICP-MS. Results are arithmetic means of at least three experiments, error bars represent SD. *** p < 0.001, ANOVA, Tukey’s multiple comparison.

Free Magnevist was taken up into glioma cells in a time- and concentration-dependent manner. As shown in Figure 3, uptake into F98 and LN229 cells was nearly linearly proportional to exposure times and concentration of gadolinium in the culture medium. DOPC-DOPE liposomes showed similar behavior, but overall uptake of the liposomal Magnevist formulation after 3 hours was 1.3 and 3 fold higher than that of free Magnevist for F98 and LN229 cells, respectively (Figure 4). Both cell lines took up cationic DOTAP-liposomes to the highest extent with 1921 ng Gd/10^6^ cells in F98 cells and 2481 ng Gd/10^6^ cells in LN229 cells after a 3 hour incubation period (Figure 5). Uptake of DOTAP-liposomes was therefore significantly higher than uptake of all other formulations, with p < 0.001 (one-way ANOVA and subsequent Tukey’s multiple comparison test, where six groups were compared separately for each cell line. LN229: R^2^ = 0.9199, 5 degrees of freedom, residual sum of squares: 1342000, degrees of freedom 21; F98: R^2^ = 0.9108, 5 degrees of freedom, residual sum of squares: 901300, 19 degrees of freedom). Interestingly, DOTAP-liposomes showed a different uptake kinetic than DOPC-DOPE and Magnevist formulations in both cell lines. As presented in Figure 4, uptake of DOTAP-liposomes reached a maximum after three hours and declined afterwards, while the other two formulations approached a plateau after 24 hours incubation time.

Furthermore, results from uptake experiments clearly show great variances in liposome uptake according to lipid composition and in part, dependency on the cell line. DOPE- and anionic Cardiolipin-liposomes transported comparable amounts of gadolinium into LN229 cells, but for F98 cells, DOPE-liposomes delivered nearly twice as much gadolinium as Cardiolipin-liposomes.

### Cell irradiation experiments

**Figure 6:**
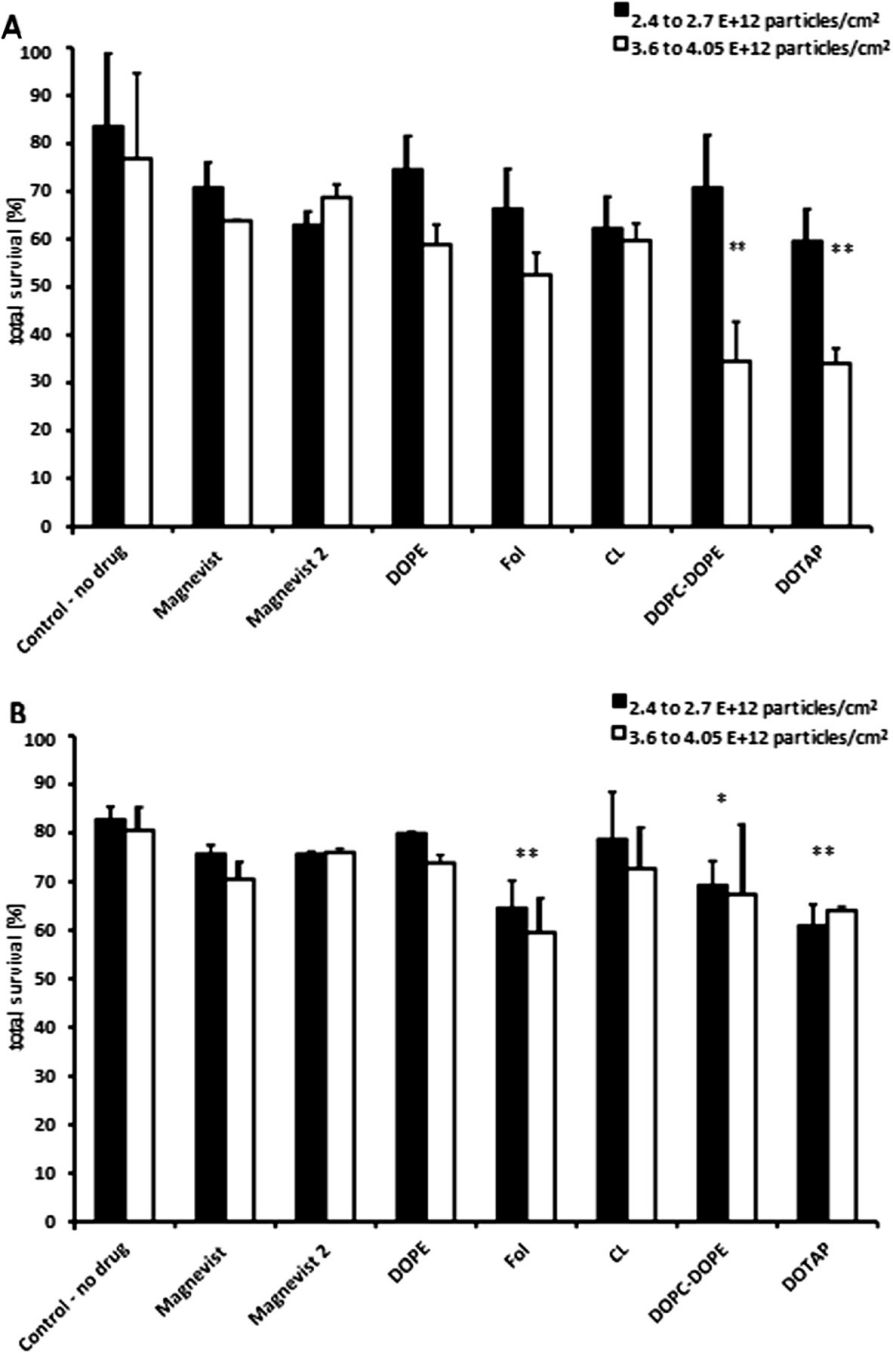
**Survival data of irradiated (A) F98 and (B) LN229 cells, 97 h after irradiation.** Cells were treated with liposomal Magnevist formulations or free Magnevist and incubated for 3 h prior to irradiation with thermal neutrons. Cells of the control group were irradiated, but contain no drug. MTT assay was performed 97 h after irradiation. Survival is based on a non-irradiated medium control without drug, results are arithmetic means of three independent experiments, error bars represent SEM. Significant differences in survival compared to control group (irradiated, medium, no drug) are marked with asterisk, * p < 0.05 and ** p < 0.01 (ANOVA, Tukey’s multiple comparison test).

Irradiation of glioma cell lines as described above led to a decreased total cell survival of 84 and 77% for F98, and 83 and 81% for LN229 cells, respectively. Treatment with ‘free’ Magnevist solution led to further decrease of survival, approximately 13% (F98 cells) and 7–10% (LN229 cells) below survival of non-irradiated control cells. Higher concentration of free Magnevist solution (Magnevist 2, cf. Figure 6) was beneficial in case of F98 cells with lower fluence, where the total survival receded to 63%. However, for higher fluence and for LN229 cells, the increased concentration of Magnevist did not improve the effect under radiation.

Application of liposomal Magnevist formulations, on the other hand, had highest effect on cell survival of both F98 and LN229 cells. While DOPE- and CL-formulations showed relatively small effect on cell survival, their application on F98 cells produced better results compared to free Magnevist under the same irradiation conditions. For LN229 cells, the outcome after treatment with DOPE- and CL-liposomes was slightly below expectations with approximately 80 and 74% survival. Nevertheless, folate -, DOPC-DOPE- and DOTAP-liposomes led to significantly lower cell survival than medium control, i.e. without drug (one-way ANOVA, subsequent Tukey’s multiple comparison test, where fluence was included as a factor, p < 0.01 (Folate - and DOTAP-liposomes), p < 0.05 DOPC-DOPE-liposomes, comparison of 14 groups, including drug-free liposomes of the different lipid compositions, degrees of freedom: 13, R^2^ = 0.9046). For F98 cells, significantly better effects compared to medium control were observed after treatment with DOTAP- and DOPC-DOPE-liposomes (one-way ANOVA, subsequent Tukey’s multiple comparison test, p < 0.01, only fluence of 3.6 to 4.05 · 10^12^ n/cm^2^, comparison of 14 groups, including drug-free liposomes of the different lipid compositions, degrees of freedom: 13, R^2^ = 0.4659). The residual sum of squares for the analysis of LN229 was 137.7, with 14 degrees of freedom. For F98, the residual sum of squares was 9241, with 44 degrees of freedom.

### Correlation between Gd-content and respective cell survival

**Figure 7:**
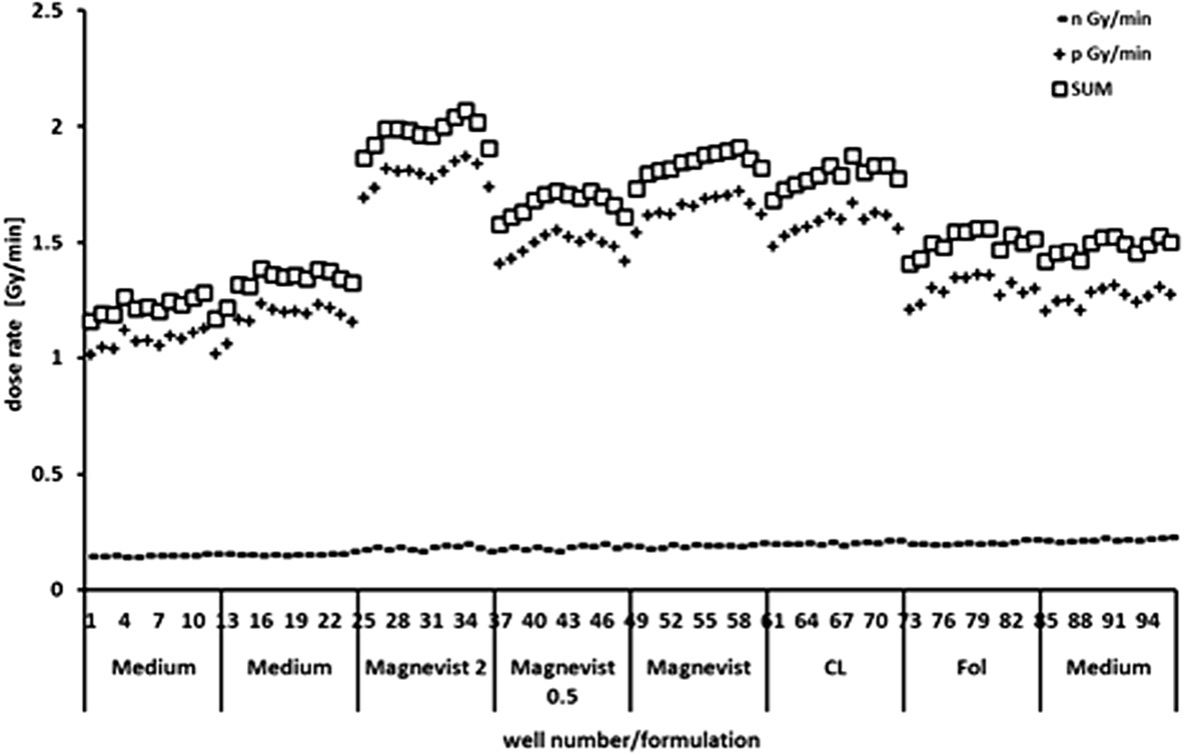
**Dose rates (neutron, photon and sum of both) on single-well level of a 96 well plate calculated via Monte Carlo simulations.** Dose profile of a 96 well plate, well number 1 to 96 from cold end to hot end of thermal column at TRIGA reactor (left to right). Wells are supplemented with 4 mg lipid/ml of respective liposomal Magnevist formulations or free Magnevist solution (rows consist of 12 wells, supplemented with the same formulation). ‘Medium’ wells contain complete growth medium only.

Gadolinium content in cells is a major factor for the overall dose and the subsequent effect on cell survival under neutron irradiation. Therefore, Monte Carlo simulations including the Gd amount per well were used to determine neutron and photon dose. Figure 7 presents the neutron and photon dose rate per minute on single-well level for irradiation of LN229 cells. In this context the term neutron dose represents all dose components arising from neutron interaction, i.e. local energy deposition from Auger and conversion electrons as well as the ^14^ N (n,p) ^14^C reaction from the nitrogen present. The diagram shows that the bigger part of the dose derives from photons generated in neutron capture events as well as photons produced in the environment of the thermal column of the radiation site. The contribution of photon self-irradiation, i.e. the dose inflicted from photons that origininate from the same well is only in the order of the natural background radiation. The dose ascribed to neutrons alone only accounts for roughly 5–10% of the overall dose (sum of neutron and photon dose). Consequently it can be said that the majority of the dose contribution arises from photon cross irradiation which is why the KERMA-approximation is not valid for simulation of dose arising from neutron capture of ^157^Gd.

**Figure 8:**
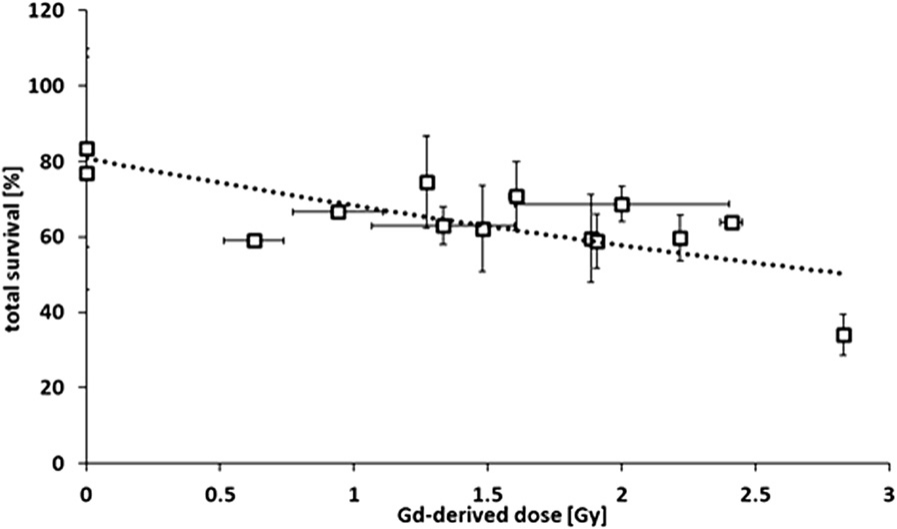
**Gd-related dose versus cell survival of F98 cells.** Correlation of Gd-derived dose (dose rate minus dose rate of control group without Gd-supplementation) versus cell survival 97 h after irradiation of F98 cells treated with the respective formulation. Results are arithmetic means of three experiments, error bars represent SD (R^2^ = 0.4671).

**Figure 9:**
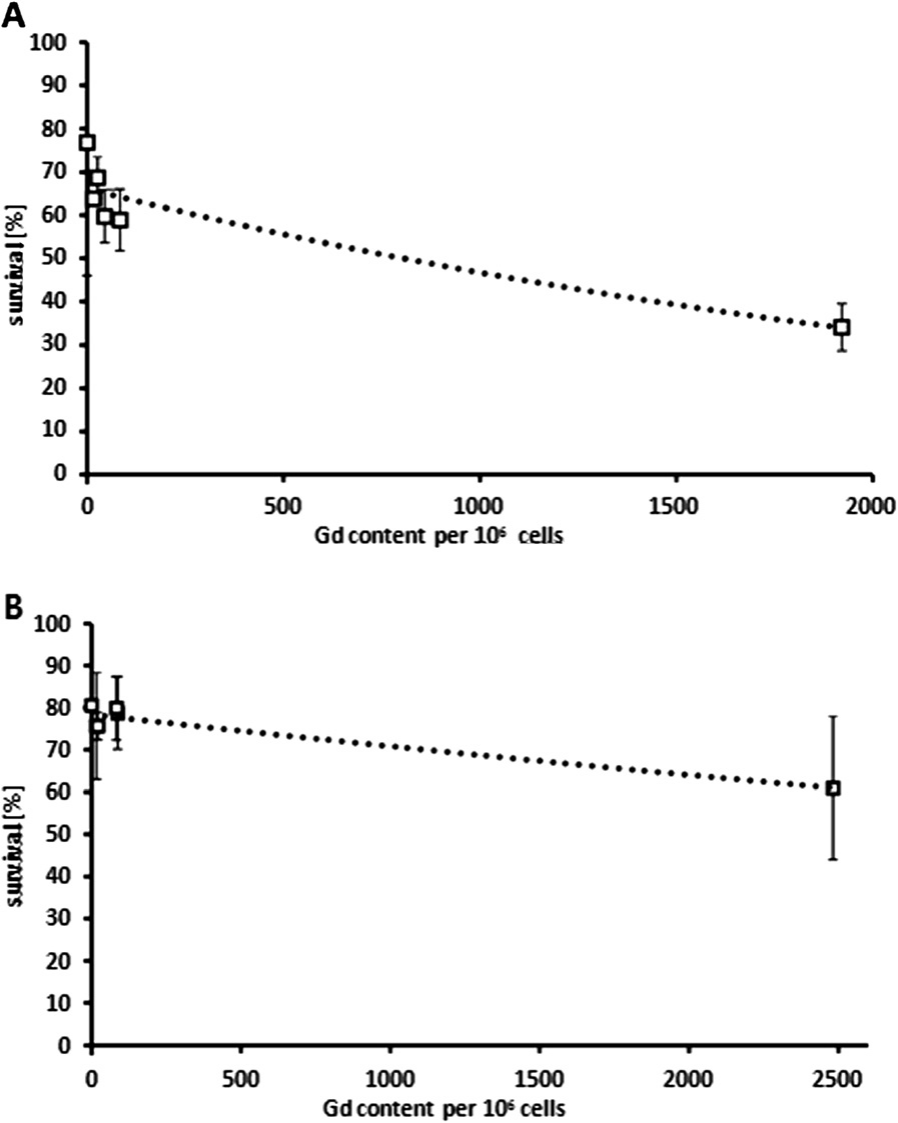
**Gd-content of (A) F98 and (B) LN229 cells versus cell survival.** Correlation of Gd-concentration in cells after three hour-incubation with liposomal Magnevist formulation or free Magnevist versus cell survival 97 h after irradiation of F98 cells with neutron fluence of 3.6 to 4.05 · 10^12^ n/cm^2^ and LN229 cells with neutron fluence of 2.4 to 2.7 · 10^12^ n/cm^2^ treated with respective formulations. Results are arithmetic means of three experiments, error bars represent SD (R^2^ = 0.8982 for F98 cells and R^2^ = 0.9274 for LN229 cells).

In the next step, cell survival and Gd-related dose on single-well level were correlated. Subtraction of simulated dose in control wells containing only medium without drug from gadolinium-containing wells resulted in Gd-related dose (both neutrons and photons generated by Gd-neutron capture event) which accounted for 13 to 32% of overall dose, i.e. 0.6 to 2.8 Gy, depending on Gd-content of the well (Figure 8). In this case, the entire amount of Gd per well is included in the calculation, irrespective of its location towards the cell, i.e. inside, after being taken up, or outside in the medium. By this means, dose from long-range gamma rays (Gd located outside the cell) and dose from Auger- and inner conversion electrons (Gd located inside the cell) after neutron capture reaction of ^157^Gd are both accounted for. However, correlation between the Gd-related dose regardless of Gd-location and cell survival was poor, with R^2^ = 0.46 and a survival decrease of 0.85% per Gy for F98 cells. Cell survival was also correlated with cellular Gd-content alone, thus neglecting the Gd concentration outside the target cells. Here, correlation of cell survival of F98 cells treated with three different liposomal formulations and free Magnevist with the respective cellular Gd-content followed an exponential relationship (R^2^ = 0.8982, survival decrease of 1%/Gy, for LN229 cells: R^2^ = 0.9274, decrease 1%/Gy) (Figure 9). However, only three of five liposomal formulations fit the correlation, while the folate-targeted and the DOPC-DOPE formulation had to be excluded. For these two formulations, the delivery of gadolinium into the cell was exceptionally small in relation to survival of cells, which in contrast decreased significantly. This anomaly might be due to different uptake pathways depending on liposome composition. In case of gadolinium for neutron capture therapy, the location of the neutron capture agent is crucial for the effect: the short-ranged Auger and conversion electrons have to reach the DNA strand in the nucleus to inflict severe lesions and lead to the desired apoptotic cell death [3]. Therefore, different endocytic pathways into the cell may offer a more satisfactory deposition of Gd inside the cell, i.e. closer to the nucleus. Thus, even small amounts of gadolinium taken up into the cell may have very high effect on cell survival.

## Discussion

Gd-NCT as alternative radiation therapy for high-grade glioma is a promising tool in cancer treatment. In contrast to BNCT, the neutron capture event produces not only short-ranged particles, such as Auger- and inner conversion electrons, but also a spectrum of longer-ranged photons. It is debatable if these photons may indeed contribute to tumor killing as some authors suggested [19-21]. According to our simulations and the geometric dimensions of our experimental set-up, the additional dose gained by longer-range photons and self-absorption would be too small, i.e. in the range of natural background radiation. For higher neutron irradiation times, there might be a beneficial effect, however, for the present study, this was not the case. Obtaining a sufficient Gd-concentration in cancer cells is therefore still a key factor in neutron capture therapy.

As described above, the estimated optimal ^157^Gd-concentration in the tumor mass was 50–200 μg/g tumor [4]. According to Dewi et al. 2013 [19] and others, previous investigations have shown that high concentrations of gadolinium are difficult to achieve and retain in tumor cells with commercially available contrast agents. Therefore, several approaches have been made to overcome the difficulty of obtaining effective Gd-concentrations in tumor mass by application of different particulate drug carriers, among others Gd-chitosan complexes, lipid nanoemulsions and gadolinium hexanedione nanoparticles [22-24]. In the present study, we have shown that variegated composite liposomes are efficient delivery agents for Gd-compound Magnevist for application in F98 and LN229 glioma cell lines. DOTAP-liposomes entrapping Magnevist were able to deliver a nearly threefold higher amount of Gd into cells than recommended (768 μg Gd/g F98 cells) and led to a highly significant decrease of cell survival. However, it has to be pointed out that Magnevist contains the natural occurring isotope mixture of gadolinium including only 15.7% of ^157^Gd, resulting in an average cross section for the mixture of 49 000 barn for thermal neutrons. Therefore, employment of ^157^Gd-enriched Gd-DTPA instead of Magnevist is expected to further enhance the radiation effect approximately fivefold. Nevertheless, three out of five Magnevist-containing liposome formulations reduced cell survival significantly, i.e. cationic DOTAP-, fusogenic DOPC-DOPE and targeted folate-liposomes. All formulations differed in uptake and reduction of cell survival, depending on liposome composition. Anionic Cardiolipin- and neutral 10% DOPE-liposomes performed only equally well as free Magnevist solution. DOTAP-liposomes were most effective in cell killing under neutron irradiation in case of F98 cells, followed by DOPC-DOPE- and folate-targeted liposomes. For LN229 cells, folate-liposomes were slightly more effective than DOTAP-formulation, but reduced survival only by additional 5% compared to the cationic liposomes.

**Figure 10:**
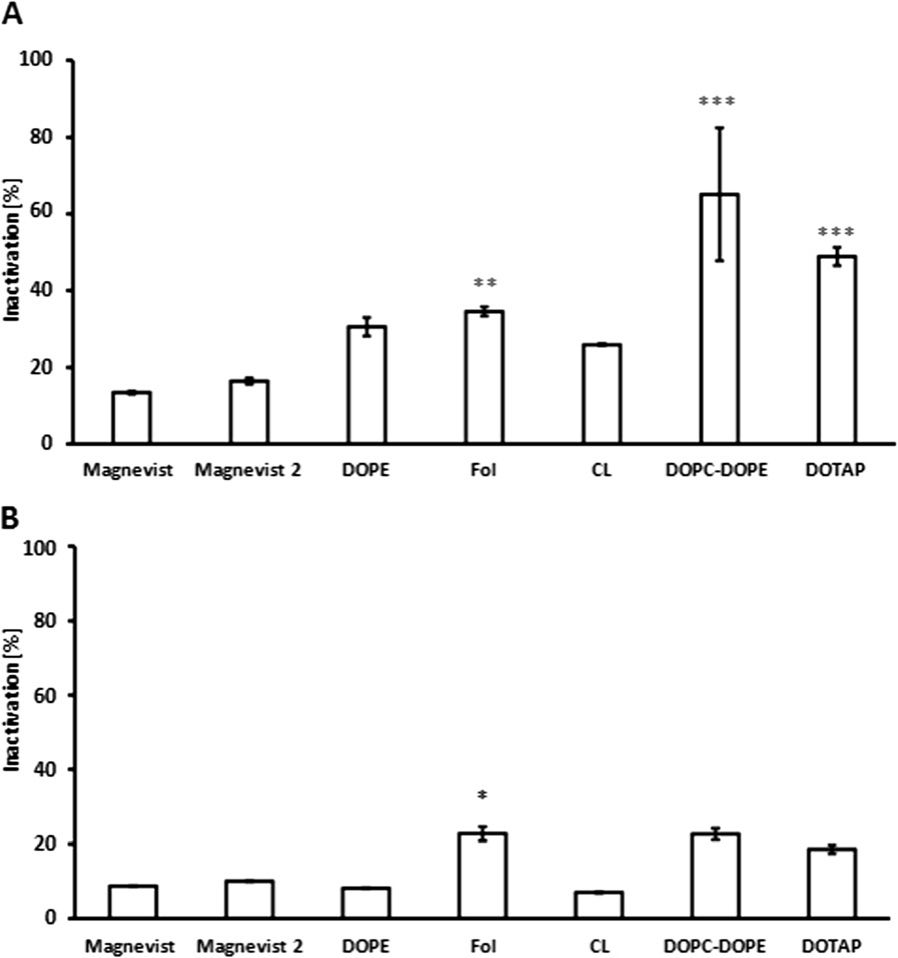
**Inactivation of glioma cells via Gd-NCT.** Inactivation of tumor cells **(A)** F98 cells, **(B)** LN229 cells, following the equation: Inactivation = 100% - Total Survival. Neutron fluences were 3.6 to 4.05 · 10^12^ n/cm^2^ for F98 cells and 2.4 to 2.7 · 10^12^ n/cm^2^ for LN229 cells. Results are arithmetic means of three independent experiments, error bars represent SEM. Total survival is based on the irradiated medium control without drug, i.e. the inactivation value shows the specific effect of the different Gd-formulations. Significance levels (ANOVA, Tukey’s multiple comparison) are * p < 0.05, ** p < 0.01 and ***p < 0.001, liposomal formulation compared to Magnevist.

The effect of the Gd-formulation alone on cellular survival is shown in Figure 10. Inactivation of tumor cells is calculated by subtraction of total survival, based on the irradiated medium control without drug, from theoretical 100% survival. Thus, the resulting value takes only the influence of the different liposomal Gd-formulations into account, thereby neglecting the ‘basal neutron irradiation’- effect provoked in all treatment groups. Although the degree of inactivation differs slightly from the effect on cell survival shown in Figure 6 - due to the use of different control groups (irradiated versus non-irradiated) - the general statement is the same: the folate-, DOTAP- and DOPC-DOPE - liposomes are the most effective Gd-formulations to inactivate both glioma cell lines. For F98 cells, these liposomal formulations lead to more than two-, three- and fivefold higher cell inactivation than the free Magnevist solution, respectively. DOPC-DOPE liposomes inactivated here as much as 65% of the cancer cells (significant results are marked with asterisks, according to results from one-way ANOVA and subsequent Tukey’s multiple comparison of 15 groups, including drug-free liposomes of the different lipid compositions, the analysis was performed separately for each cell line and for each fluence. F98 cells, 3.6 to 4.05 · 10^12^ n/cm^2^: R^2^ = 0.8582, residual sum of squares: 1795, 37 degrees of freedom; LN229 cells, 2.4 to 2.7 · 10^12^ n/cm^2^: R^2^ = 0.7352, residual sum of squares: 772, 33 degrees of freedom). For LN229 cells, the effect was lower, but still in the range of 23% inactivation through the folate-targeted liposomes giving significantly better results than the free Magnevist solution. With the additional effect from the neutron irradiation, i.e. in comparison with non-irradiated control cells, the cellular survival can be reduced by 40% in case of LN229 cells via administration of folate-targeted Gd-formulation and even by 66% for F98 cells treated with Gd-containing DOTAP-liposomes.

A likely explanation of high uptake and high effectiveness of DOTAP-liposomes is the electrostatic interaction between cationic liposome and anionic cell membrane, facilitating the binding step in endocytosis [25]. Moreover, DOTAP liposomes seem to enter the cell by a different uptake pathway than DOPC-DOPE liposomes, apparent in different uptake kinetics of the two formulations. Accordingly, the uptake pathway also attributes to cell killing effectiveness of the liposomal Magnevist in addition to the total extent of liposome uptake. DOPC-DOPE- and folate-targeted liposomes as well were more effective than other liposome formulations delivering higher amounts of Gd into cells, suggesting that different uptake modalities of the formulations may be responsible for their different efficacy. In case of DOPC-DOPE liposomes, the phenomenon can be ascribed to facilitated escape of the liposome from the endosome through conformational change of DOPE under acidic conditions – a feat often exploited for gene delivery purposes [18,26]. Once released into the cytosol, Gd-DTPA may easily diffuse to the nucleus and reside in proximity of the DNA instead of being trapped in lysosomes or vacuoles. For folate-targeted liposomes, their uptake pathway is still under investigation. It remains unclear whether there is indeed an alternative pathway from clathrin independent carriers or caveolin-mediated uptake instead of lysosomal degradation, namely leading to the endoplasmic reticulum in proximity to the nucleus - from where high-linear energy transfer (LET) particles would have better access to cellular DNA [16,27-29]. The results from cell irradiation experiments suggest that the extracellular Gd-concentration that was comparable for all formulations does have very little influence on cell survival compared to the intracellular concentration. Nevertheless, long range gamma rays may in part be reabsorbed by Gd present in tumor tissue and add to the overall dose (‘self-absorption’), but the effect is very small. Therefore, the accumulation of Gd inside the cell seems to be responsible for the major part of DNA lesions leading to cell death. Correlation of extracellular Gd-amount to cell survival was very poor, but tendencies were distinguishable as higher Gd-concentrations led to lower cell survival. In contrast, cellular Gd-concentration plotted against cell survival showed good correlation. However, discrepancy between cellular Gd-amount and cell survival was obvious in case of DOPC-DOPE and folate-liposomes, as described above, suggesting that intracellular distribution of Gd plays a prominent role in Gd-NCT and has to be taken in account when planning NCT-treatment.

Interestingly, FR 1-positive LN229 and non-FR 1-expressing F98 cells likewise took up folate-targeted liposomes. These findings are similar to Moret et al. 2013 [30] who found also a slightly higher uptake of folate-bearing liposomes into FR 1-negative A549 cells compared to non-targeted liposomes, whereas uptake of folate-liposomes was enhanced approximately twofold in FR 1-positive KB cells. In our study, enhanced uptake of targeted liposomes into LN229 was correspondingly 1.25 to 2-fold higher than for non-targeted formulations, with the exception of cationic DOTAP-liposomes. In comparison to Lee and Low 1995 [31], who reported 45-fold enhancement, this increase is relatively small. However, one has to bear in mind that overall FR 1 expression on gliomas or other brain tumors is usually very low, thus leading to only slightly enhanced uptake in FR positive cells [14]. Nevertheless, Saul et al. 2003 [13] have found that even in brain tumors showing low FR 1 expression, folate-targeted drug carriers could be very useful. After the density of folate-moieties on the carrier surface had been optimized for low-FR-1 expression C6 glioma cells, they noted a considerable intracellular increase of the loaded drug, doxorubicin, while uptake into non-FR 1 expressing E9 cells was still low. As a consequence, differentiation between FR 1- positive tumor and FR 1-negative surrounding normal cells may be achieved even at low FR 1-receptor levels, thereby increasing effectiveness of radiation enhancers under neutron capture therapy.

Anionic liposomes containing Cardiolipin as marker lipid were taken up to a lower extent than cationic or folate-liposomes and led to smaller decrease in cell survival. As a lipid involved in apoptosis, we expected higher effect on cell killing after uptake into F98 and LN229 cells and maybe even higher uptake due to the cell-innate character of the lipid. Unfortunately, the formulation did not achieve satisfactory results under neutron irradiation. For one part, low entrapment efficiency and therefore lower transport of Gd into cells may be responsible for weak performance under neutron capture conditions. On the other hand, the even lower delivery of Gd by DOPC-DOPE liposomes did not diminish their effectiveness for both cell lines. However, if cell debris containing Cardiolipin as apoptosis marker is usually taken up by glia cells and sorted into lysosomes, glioma cells are likely to act likewise with liposomes containing the same lipid. Gd in lysosomes may then be located too far away from the cellular nucleus for efficient NCT. These findings suggest that indeed the intracellular distribution of the Gd has higher impact on the success of the therapy than the simple amount of Gd delivered into target cells.

As was shown by Monte Carlo simulations, overall dose on single-well level was mainly dictated by photons derived from neutron capture events or from environment of the thermal column. Doses varied from 4 and 6 Gy (control group) to approximately 6 and 9 Gy (Magnevist 2 group) depending on Gd-content, for low and high neutron fluence, respectively. The dose in 10^6^ cells was calculated according to the up taken amount of gadolinium delivered by the respective liposomes formulation, minus the dose inflicted by the environment such as the neutron capture of nitrogen and the surrounding photons (from control group). Consequently, the dose on the cellular level was very small (0.001 to 0.3 Gy), but as observed from correlation of dose to cell survival, of high importance for the irradiation effect and differences between liposome formulations.

## Conclusions

Gadolinium-containing composite liposomes presented here proved to be effective drug delivery agents for neutron capture therapy *in vitro*. The application of different liposomal formulations of FDA-approved MRI contrast agent Magnevist led to significantly lower cell survival of glioma cells compared to non-encapsulated Gd-DTPA for three out of five new liposome compositions (DOTAP-, DOPC-DOPE-, folate-liposomes), even without ^157^Gd enrichment. For folate-targeted liposomes, specificity for low receptor expression in brain cancer cells may be further optimized via addressing the folate-density on liposome surface.

Furthermore, liposome composition and specific uptake properties have high impact on irradiation effect, i.e. cell killing under neutron irradiation, probably due to different endocytic pathways and subsequent spatial orientation on cellular level. The proximity of Gd-atoms to cellular DNA was proven to be crucial for infliction of lethal damage. Investigations of liposomal uptake mechanisms and intracellular trafficking according to lipid composition are therefore key points for further studies and optimization of ‘Gd-loading’ of tumor cells before neutron irradiation.

## Abbreviations

BNCT: Boron neutron capture therapy
CL: Cardiolipin
DOTAP: 1,2-Dioleoyl-3-trimethylammonium-propane
DOPE: 1,2-Dioleoyl-*sn*-glycero-phosphoethanolamine
DOPC: 1,2-Dioleoyl-sn-glycero-3-phosphocholine
FDA: U.S. food and drug administration
Fol-2000PEG-DSPE: 1,2-distearoyl-*sn*-glycero-3-phosphoethanolamine-N-[folate(polyethylene glycol)-2000]
FR 1: Folate receptor 1/ folate receptor α
LET: Linear energy transfer
MRI: Magnetic resonance imaging
NCT: Neutron capture therapy
